# Some Remarks upon the Functions of the Nervous System, in Connection with the Views of Sir Chas. Bell, Dr. Marshall Hall, and Dr. Bennett Dowler

**Published:** 1853-01

**Authors:** 


*Some Remarks upon the Functions of the Nervous System, in connection with the views of Sir Chas. Bell, Dr. Marshall Hall, and Dr. Bennett Dowler.* By B. F. Taylor, M. D., La.

It is known to the student of physiology, that Sir Chas. Bell long ago announced his views in reference to the functions of the spinal cord, and endeavored to prove that it consisted of *four sets of fibres*—each of which was made to perform a separate function, though intimately associated with each other.

1st. A *sensory* bundle.
2d. A *motor set.*
3d. A set of *excitor* or centripetal fibres.
4th. A *motor* or centrifugal set.

The first and third are united in the posterior, the second and fourth in the anterior column of the spinal marrow. In other words, the *anterior for motion*, the *posterior for sensation*, and the *middle column for respiration.*

After the inception of this new theory, physiologists found it exceedingly difficult to trace the course of the fibres within the spinal cord; in consequence of which, Sir Charles Bell's views began to be distrusted. In the mean time, cases were constantly occurring, when a portion of one of the columns was found almost entirely destroyed by disease, with a corresponding loss of function.

Whilst the merits and demerits of this new discovery were being warmly discussed, by British and Continental Physiologists, a new star arises, in the person of Dr. Marshall Hall, who was destined to shed a brilliant, but an ephemeral light, upon the profession—but to give place to one far greater in magnitude and brilliancy, in the person of an eminent *savan*, whose genius and discoveries are destined to reflect additional lustre upon the American name and physical science wherever it is cultivated.

Dr. Marshall Hall contends, that the nerves of the spinal column have a *fourfold set of functions—a double set of excito-motor, and of sensori-volitional nerves*—and explains every phenomenon connected therewith, as purely reflex in its character. It cannot be denied but that this theory is surrounded with a great deal of mystery, since the edtior of the London Lancet, in his rapturous support of the doctrine, was compelled to announce his conviction, that "not half a dozen of the members of the Royal College of Physicians could comprehend his peculiar views; and that he was an hundred years beyond his contemporaries," &c. Looking through a *Hall-medium*, it is not to be marveled that Mr. Wakly should still continue to support those views, since it is shrewdly suspected that Marshall Hall himself controls that able and influential Journal.

Dr. Bennett Dowler, with a view of testing the truth of Sir Charles Bell's and Marshall Hall's views, has made a series of experiments upon the great Saurian, whose tenacity of life is greater than that of any known animal, and who exhibits the phenomena of reflex action upon a much greater scale than frogs—which demonstrate the fallacy of a "fourfold set of functions," in opposition to which, very satisfactory and nicely conducted experiments are adduced.

In reviewing Dr. Dowler's paper, the editor of the British and Foreign Medico-Chirurgical Review, the most able reviewer in Europe, had the manliness and moral courage to come out boldly and renounce his adhesion to the "fourfold system of nerves," "now generally admitted," says he, "amongst well informed physiologists, such having, *as we now believe, no real existence in nature.*" An admission, Dr. Hester judiciously remarks, which "forms an epoch in scientific progress, because with certain individuals it will weigh more than any amount of *demonstration, intuition*, or possibly *revelation* itself."

Dr. Dowler's discovery of a *diffused sensorium*, has furnished a key to the hidden recesses of the nervous system. The adaptation of those views to therapeutic medicine is most strikingly and beautifully illustrated in man's first ingress into this breathing world. The first respiratory effort of the new born infant is most vigorously performed when the cool air comes in contact with its general surface. Accoucheurs avail themselves of this important fact; hence the utility of slapping, frictions, and the application of cold water, in more effectually exciting the respiratory movements. In the treatment of asphyxia by pouring, hysteria, &c., the alternate application of heat and cold, is most powerfully manifest in restoring these movements. All of these phenomena are in harmony with nature's laws,—written upon the nervous system,—and the application of the doctrine of a *diffused sensorium.*

January, 1852.—*N. O. Med. and Surg. Journal.*

